# Effectiveness of four vaccines in preventing SARS-CoV-2 infection in Almaty, Kazakhstan in 2021: retrospective population-based cohort study

**DOI:** 10.3389/fpubh.2023.1205159

**Published:** 2023-06-07

**Authors:** Dilyara Nabirova, Roberta Horth, Manar Smagul, Gaukhar Nukenova, Aizhan Yesmagambetova, Daniel Singer, Alden Henderson, Alexey Tsoy

**Affiliations:** ^1^Division of Global Health Protection in Central Asia, United States Centers for Disease Control and Prevention, Almaty, Kazakhstan; ^2^Central Asia Field Epidemiology Training Program, Asfendiyarov Kazakh National Medical University, Almaty, Kazakhstan; ^3^Scientific and Practical Center of Sanitary-Epidemiological Examination and Monitoring, Branch of the National Center for Public Health, Almaty, Kazakhstan; ^4^Committee of Sanitary and Epidemiological Control, Ministry of Healthcare, Astana, Kazakhstan; ^5^Division of Global Health Protection, United States Centers for Disease Control and Prevention, Atlanta, GA, United States; ^6^Office of the Minister, Ministry of Healthcare, Astana, Kazakhstan

**Keywords:** COVID-19, vaccine effectiveness, breakthrough infection, SARS-CoV-2, QazVac, Kazakhstan

## Abstract

**Background:**

In February 2021 Kazakhstan began offering COVID-19 vaccines to adults. Breakthrough SARS-CoV-2 infections raised concerns about real-world vaccine effectiveness. We aimed to evaluate effectiveness of four vaccines against SARS-CoV-2 infection.

**Methods:**

We conducted a retrospective cohort analysis among adults in Almaty using aggregated vaccination data and individual-level breakthrough COVID-19 cases (≥14 days from 2nd dose) using national surveillance data. We ran time-adjusted Cox-proportional-hazards model with sensitivity analysis accounting for varying entry into vaccinated cohort to assess vaccine effectiveness for each vaccine (measured as 1-adjusted hazard ratios) using the unvaccinated population as reference (*N* = 565,390). We separately calculated daily cumulative hazards for COVID-19 breakthrough among vaccinated persons by age and vaccination month.

**Results:**

From February 22 to September 1, 2021, in Almaty, 747,558 (57%) adults were fully vaccinated (received 2 doses), and 108,324 COVID-19 cases (11,472 breakthrough) were registered. Vaccine effectiveness against infection was 79% [sensitivity estimates (SE): 74%–82%] for QazVac, 77% (SE: 71%–81%) for Sputnik V, 71% (SE: 69%–72%) for Hayat-Vax, and 70% (SE: 65%–72%) for CoronaVac. Among vaccinated persons, the 90-day follow-up cumulative hazard for breakthrough infection was 2.2%. Cumulative hazard was 2.9% among people aged ≥60 years versus 1.9% among persons aged 18–39 years (*p* < 0.001), and 1.2% for people vaccinated in February–May versus 3.3% in June–August (p < 0.001).

**Conclusion:**

Our analysis demonstrates high effectiveness of COVID-19 vaccines against infection in Almaty similar to other observational studies. Higher cumulative hazard of breakthrough among people ≥60 years of age and during variant surges warrants targeted booster vaccination campaigns.

## Introduction

From March 2020 to December 17, 2021 in Kazakhstan, over one million people were diagnosed with coronavirus disease 2019 (COVID-19); ~18,000 died (1). Vaccines have become a key tool for reducing COVID-19-associated morbidity and mortality and preventing transmission of SARS-CoV-2. In February 2021, Kazakhstan began offering COVID-19 vaccines to adults aged 18 years and older. By December 9, 2021, 71% of adults (~eight million people) had received at least two doses of vaccines, including Sputnik V (Gam-COVID-Vac, Gamaleya Research Institute of Epidemiology and Microbiology, Russia), QazVac (QazCovid-in, Research Institute for Biological Safety Problems, Kazakhstan), CoronaVac (Sinovac Biotech, China), Sinopharm (BBIBP-CorV, Beijing Institute of Biological Products, China) and Hayat-Vax (BBIBP-CorV, G42 Healthcare, United Arab Emirates) (2).

Of the five vaccines available in Kazakhstan, two (CoronaVac and Sinopharm) were listed for emergency use globally by the World Health Organization (WHO) by end of 2021. The WHO pre-qualifies, and lists vaccines based on several guidelines (3) including good manufacturing practice and >50% vaccine efficacy in preventing SARS-CoV-2 outcomes in clinical trials (4, 5). Among the WHO listed vaccines, vaccine efficacy against symptomatic disease in randomized-controlled trials was 51%–84% for CoronaVac, 79% for Sinopharm, 63%–76% for AstraZeneca, and >90% for Moderna and Pfizer–BioNTech vaccines (4–6).

Although not listed by the WHO, Sputnik V and QazVac vaccines have received emergency use authorization from the Kazakhstan Ministry of Healthcare. Both have >90% efficacy and safety in phase II and III clinical trials (7–10). Additionally, Sputnik V demonstrated 79%–86% effectiveness against infection and 85%–93% effectiveness against COVID-19 related death in several observational studies outside of clinical trials (real-world).

Increasing breakthrough COVID-19 infections among vaccinated people raised concerns about the real-world effectiveness of available vaccines. This was especially concerning in Kazakhstan where vaccine hesitancy was high, and there was distrust in the newly developed vaccines offered in the country. By the end of 2021, there were no real-world effectiveness studies on QazVac vaccine and limited data on the effectiveness of Sputnik V. Understanding how vaccines are performing outside of clinical trials is important for healthcare system preparedness. Additionally, it is important to continually monitor effectiveness of vaccines in different geographic regions, time periods, populations, to best inform targeted local mitigation measures that are needed for a novel virus that is continually mutating.

Policy makers in Kazakhstan needed data to create evidence-based vaccine guidelines, including policies for booster vaccine doses for different populations. We aimed to estimate daily hazard of breakthrough SARS-CoV-2 infection among vaccinated people in Almaty, to identify groups at higher risk for breakthrough infection, and to evaluate the overall effectiveness of four vaccines against infection. Vaccine efficacy and effectiveness studies can be costly and resource intensive, we therefore aimed to demonstrate an approach to assess real-world effectiveness using existing data collected routinely by the public health system.

## Materials and methods

### Study design

We conducted a retrospective, observational population-based cohort study of the effectiveness of four different vaccines against SARS-CoV-2 infection among the entire adult population (18 years or older) of Almaty, Kazakhstan between February 1, and September 1, 2021. Sinopharm vaccine was excluded from this assessment because only 20 participants received this two-dose vaccine during this period in Almaty. Almaty is the economic capital and largest city of Kazakhstan with the population of 1,928,000. Adults constituted 68% (1,313,040) of Almaty and ~10% of the national adult population in 2021 (11).

### COVID-19 surveillance system

All healthcare providers in Kazakhstan are mandated to report all COVID-19 cases and vaccines into national registries. Our study uses the routinely collected data from these national registries. The national case-based COVID-19 surveillance system in Kazakhstan is managed by the Ministry of Healthcare. Everyone tested for COVID-19, regardless of test result, is registered in the system. COVID-19 PCR testing is provided free of charge to all persons with symptoms and all contacts of cases. This testing is conducted at all primary health care facilities, in home-based visits, and in hospitals. COVID-19 testing is also widely available at private laboratories in Kazakhstan for a fee. All persons traveling internationally and unvaccinated people in different settings were required to test. Home-based COVID-19 antigen testing was not available during the period covered by this study. In addition, the COVID-19 positivity rate during this study ranged from 5% to 7%.

During the study period COVID-19 vaccination was also provided free of charge to all adults 18 years and older. All vaccinations are registered in a national vaccine registry. Adverse effect experience in the first 3 days after vaccination are registered.

Community mitigation strategies in place during this time were masking, contact tracing, isolation and quarantine of cases and contacts which was monitored by a nationwide mobile phone app linked to peoples’ national identification number.

### Key definitions

A breakthrough COVID-19 infection was defined as having confirmed or probable COVID-19, including people with asymptomatic disease, ≥14 days after receiving the 2nd dose of a COVID-19 vaccine.

A confirmed COVID-19 case was defined as the detection of SARS-CoV-2 ribonucleic acid (RNA) in a respiratory specimen collected from a person aged ≥18 years, including those with asymptomatic disease.

A probable COVID-19 case was a patient who met clinical criteria (chest imaging suggesting COVID-19, anosmia, ageusia in the absence of any other identified cause), did not have a positive SARS-CoV-2 PCR test, and was a contact of a probable or confirmed case or linked to a COVID-19 cluster.

Fully vaccinated was defined as having received two COVID-19 vaccine doses (there were no single dose COVID-19 vaccines offered in Kazakhstan). Unvaccinated was defined as never having received any COVID-19 vaccine.

People who received only 1 dose or got COVID-19 <14 days after their second dose are considered not fully immune and are censored (although not excluded) from analysis at date of 1st vaccine dose.

We classified people into four groups. People who were:

1. unvaccinated (never received any COVID-19 vaccine) and diagnosed with COVID-19,
2. unvaccinated (never received any COVID-19 vaccine) and not diagnosed with COVID-19,
3. fully vaccinated (two doses) and diagnosed with breakthrough COVID-19, and.
4. fully vaccinated (two doses) and not diagnosed with breakthrough COVID-19.

### Study population

**Figure 1:**
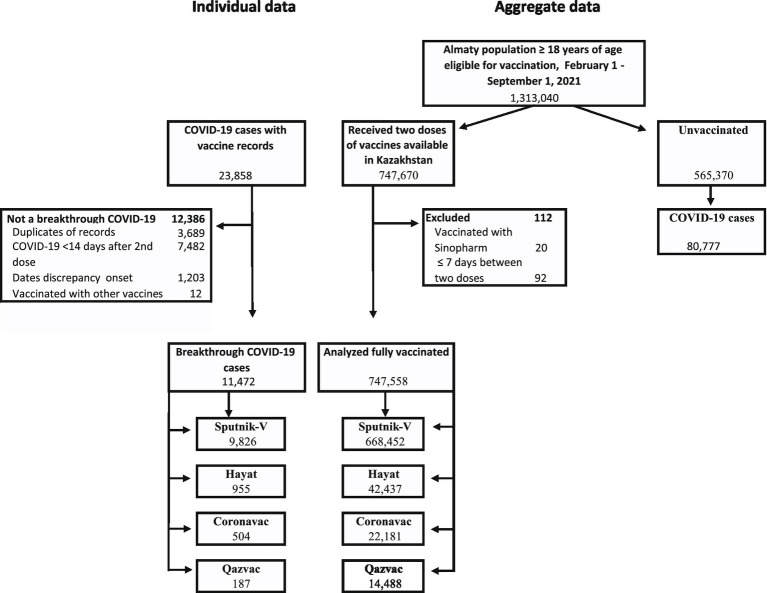
Flow of participants in a study of effectiveness of four vaccines in preventing COVID-19 in Almaty, Kazakhstan, 2021.

Our analysis included Almaty residents ages 18 years and older. The following people were excluded from the time-dependent Cox proportional hazards regression analysis (Figure 1):

1. People whose records were inconsistent with national vaccination guidelines (e.g., second dose was <7 days of the first dose or they had COVID-19 diagnosis <25 days before their second dose).
2. People who received the Sinopharm vaccine (only 20 people).

Data on history of COVID-19 prior to the study period was not available; therefore, we do not exclude based on prior disease.

### Data sources

Individual-level data on COVID-19 cases among anyone who had ever received a COVID-19 vaccine from February 1 to September 1, 2021 were provided by the Emergency Operational Center (EOC) of the National Center of Public Health of the Ministry of Healthcare in Kazakhstan. Data on all COVID-19 cases from testing centers, laboratories, hospitals, and healthcare providers are collected by the EOC. This clinical record dataset included age, comorbidities, dates of COVID-19 vaccination, vaccine name, date of SARS-CoV-2 PCR test result or diagnosis, hospitalization, death, and disease severity. Disease severity was based on the WHO Scale adapted by the Kazakhstan clinical protocol of diagnosis and treatment. People with critical disease had acute respiratory distress, those with severe disease had severe pneumonia, those with moderate disease had pneumonia, and those with mild disease did not have pneumonia. Data aggregated per day on SARS-CoV-2 polymerase chain reaction (PCR) testing, which included the number of people testing positive per day for the same period, were also provided.

Aggregated data on vaccination were received from the Salidat Kairbekova National Research Center for Health Development of the Ministry of Healthcare in Kazakhstan. Variables in this dataset were given to us aggregated by date and included the number of people vaccinated per day per age groups (18–39, 40–59, and ≥60 years) and vaccine type.

### Statistical analysis

Analyses were performed using R statistical software, version 4.0.3 (R Foundation for Statistical Computing). We calculated and plotted attack rates for the vaccinated and unvaccinated populations for each day of our analysis for each vaccine type. As is commonly used in vaccine effectiveness analysis (12–16), we fitted standard time-dependent Cox proportional hazards regression models using the coxph function from the Survival package (the specific function code was: coxph(Surv(timestart, timestop, covidstatus)) ~ vaccinestatus, data = timedependentdataframe, ties = “breslow”) to calculate hazards ratios for COVID-19 by vaccination status and vaccine type (17–21). Vaccination status was a time-dependent covariate that changed to partially vaccinated when the person received their 1st dose and to fully vaccinated at 14 days after their 2nd dose. We conducted analysis for all vaccines, and separately by vaccine type.

The time of entry into the model was set at February 1, 2021, when COVID-19 vaccines became available in Almaty. Participants were retrospectively followed until the end of the study period or until they were censored (max days of follow-up was 212 days). The entire population of adults ≥18 years old in Almaty (*N* = 1,313,040) was classified into the unvaccinated group at-risk for COVID-19 group. People contributed person-time to the unvaccinated population until they either received a COVID-19 diagnosis or a COVID-19 vaccine. Persons receiving a COVID-19 diagnosis were censored at their time of diagnosis. After people received their first vaccine, they did not contribute person-time to either population (vaccinated or unvaccinated) from their first dose to 14 days after their second dose after which they contributed person-time to the vaccinated population. We performed sensitivity analysis by changing the dates at which people entered the vaccinated population (at first dose, second dose, second dose +7 days or second dose +14 days) and the dates of definition of a COVID-19 breakthrough case (after second dose, second dose +7 days or second dose +14 days). We tested for the proportional hazards (PH) assumption using Schoenfeld residuals, which tests whether there is a relationship between residuals and time, using cox.zph function from the survival package in R (16). All models met this assumption. We also conducted sensitivity analysis adjusting for the daily cumulative percentage of people ages 60 and above that were vaccinated and the daily percent positivity of COVID-19 testing. We provide high and low sensitivity estimates for our results to express the uncertainty more accurately around estimates.

We separately calculated crude (unadjusted) vaccine effectiveness for each vaccine as per the WHO guidance (22, 23).

We also estimated the crude (unadjusted) number of COVID-19 cases prevented by multiplying the number of vaccinated people by the overall incidence among the unvaccinated population and subtracting the number of breakthrough cases.

Lastly, we conducted sub analysis on the vaccinated population. We calculated the cumulative hazard (the estimate of the expected number of COVID-19 cases that has been observed for the given amount of time) of getting COVID-19 using survfit() and plotted Kaplan–Meyer cumulative hazard curves using ggsurvplot() function and by vaccine name, age group, and month. The log-rank test was used to test difference in hazard curves (19).

## Results

From February 22 to September 1, 2021, in Almaty, 747,558 residents aged ≥18 years were fully vaccinated against COVID-19 with Sputnik-V (668,452), Hayat-Vax (42,437), CoronaVac (22,181), or QazVac (14,488) and met inclusion criteria. During this time, Almaty had 108,324 probable and confirmed COVID-19 cases among people ≥18 years old, including 23,858 cases among people with at least one COVID-19 vaccination record (Figure 1). Of these 11,472 were fully vaccinated and met criteria for breakthrough SARS-CoV-2 infection.

Among fully vaccinated people with breakthrough SARS-CoV-2 infections (Table 1), 24% were ≥60 years old, 11% had a known comorbidity and presence of comorbidity was unknown for 72%. The median interval between vaccine doses was 21 days (range 8–166). The median interval between second dose and breakthrough infection was 59 days (range 14–178). Disease was classified as critically severe for 0.7% and severe for 6.8% of cases, and 29% were hospitalized. There were 120 deaths (1%).

**Table 1:** Characteristics of fully vaccinated people with SARS-CoV-2 breakthrough infection, Almaty, Kazakhstan, 2021.

Characteristics	*N* = 11,472	% or (range)
**Sex**		
Female	6,923	60.3
Male	4,549	39.7
**Age, median (range)**	45	(range 18 to 96)
18–39 years	4,360	38.0
40–59 years	4,351	37.9
≥60 years	2,761	24.1
**Had a comorbidity**		
No	1,892	16.5
Yes	1,271	11.1
Unknown	8,309	72.4
**Fully vaccinated**		
Sputnik V	9,826	85.7
Hayat-Vax	955	8.3
QazVac	187	1.6
CoronaVac	504	4.4
**COVID-19 waves in Kazakhstan**		
February 1–June 30, 2021	402	3.5
July 1–31, 2021	2,335	20.4
August 1–September 1, 2021	8,735	76.1
**PCR-identified cases**		
PCR −^a^	560	4.9
PCR +	10,912	95.1
**Interval between 1st and 2nd dose, median (range)**	21	(range 8 to 166)
8–14 days	259	2.3
15–19 days	395	3.4
20–25 days	8,449	73.6
26–46 days	1,906	16.6
47–166 days	463	4.0
**Days since fully vaccinated to disease onset, median (range)**	59	(range 14 to 178)
**COVID-19 severity** ^b^		
Critical disease	78	0.7
Severe disease	776	6.8
Moderate disease	2,154	18.8
Mild disease	8,464	73.8
**Hospitalized**		
No	8,146	71.0
Yes	3,326	29.0
**Number of hospitalizations** ^c^		
One hospitalization	3,285	98.8
Two hospitalizations	41	1.2
**Length of hospitalization in days, median (range)** ^c^	9	(range 1 to 35)
**Outcome**		
Recovered or transferred	11,352	99.0
Died	120	1.0

a PCR negative cases were classified as probable COVID-19 cases using WHO definition, “clinical criteria and is a contact of a probable or confirmed case, or linked to a COVID-19 cluster.”

b WHO Scale adapted by the Kazakhstan clinical protocol of diagnosis and treatment (Coronavirus infection COVID-19 in adults).

c Subset of persons having been hospitalized.

Vaccination began on February 1, 2021, for Sputnik, April 25 for QazVac, May 3 for Hayat-Vax and June 6 for CoronaVac. During the study period, the adjusted vaccine effectiveness for all four vaccines was 76.4% [low and high sensitivity percent range (SE):71.1%–80.0%] (Table 2). Adjusted vaccine effectiveness varied by vaccine and was 77.0% (SE: 71.4%–80.8%) for Sputnik V, 78.6% (SE: 74.2%–82.0%) for QazVac, 71.2% (SE: 69.1%–72.4%) for Hayat-Vax, and 69.5% (SE: 64.7%–71.5%) for CoronaVac (*p* < 0.001). Vaccine effectiveness was similar across models that adjusted for time, daily cumulative proportion of vaccinated people that were ≥60 years old, and daily COVID-19 positive test rate (Appendix A: Supplementary Table). The proportion of people aged ≥60 years old increased in April and stabilized thereafter (Appendix B: Supplementary Figure).

**Table 2:** Vaccine effectiveness against SARS-CoV-2 infection of four COVID-19 vaccines in Almaty, Kazakhstan, 2021.

Category	Fully vaccinated, *N*	% aVE	95% CI	Sensitivity analysis				% cVE	95% CI
				Low	95% CI	High	95% CI		
**Vaccination status**									
Vaccinated	747,558	76.4^a^	(75.9-76.9)	71.1	(70.5–71.6)	80.0	(79.6–80.4)	89.7	(89.5–89.9)
Not vaccinated	565,370	Ref						Ref	
**Vaccine name**									
QazVac	14,488	78.6^a^	(75.3-81.4)	74.2	(70.2–77.7)	82.0	(79.2–84.4)	85.8	(83.6–87.7)
Sputnik-V	668,452	77.0^a^	(76.5-77.5)	71.4	(70.8–72.1)	80.8	(80.4–81.2)	90.4	(90.2–90.6)
Hayat-Vax	42,437	71.2^a^	(69.3-73.0)	69.1	(67.1–71.0)	72.4	(70.6–74.1)	73.2	(71.4–74.8)
CoronaVac	22,181	69.5^a^	(66.7-72.0)	64.7	(61.5–67.7)	71.5	(68.9–73.9)	71. 1	(68.5–73.5)
Not vaccinated	565,370	Ref						Ref	

aVE, adjusted vaccine effectiveness, adjusted for time and vaccination status; CI: confidence interval. Low: day of contribution of person-time to vaccinated population begins on day of 1st dose. High: day of contribution of person-time to vaccinated population begins 14 days after 2nd dose. cVE: crude vaccine effectiveness; persons partially vaccinated (*n* = 7,482) and those with a case registered before February 22, 2021 (*n* = 2018) were excluded from the unvaccinated reference group.

a Time-dependent Cox-proportional hazards log-rank test *p*-value for each model was <0.001.

**Figure 2:**
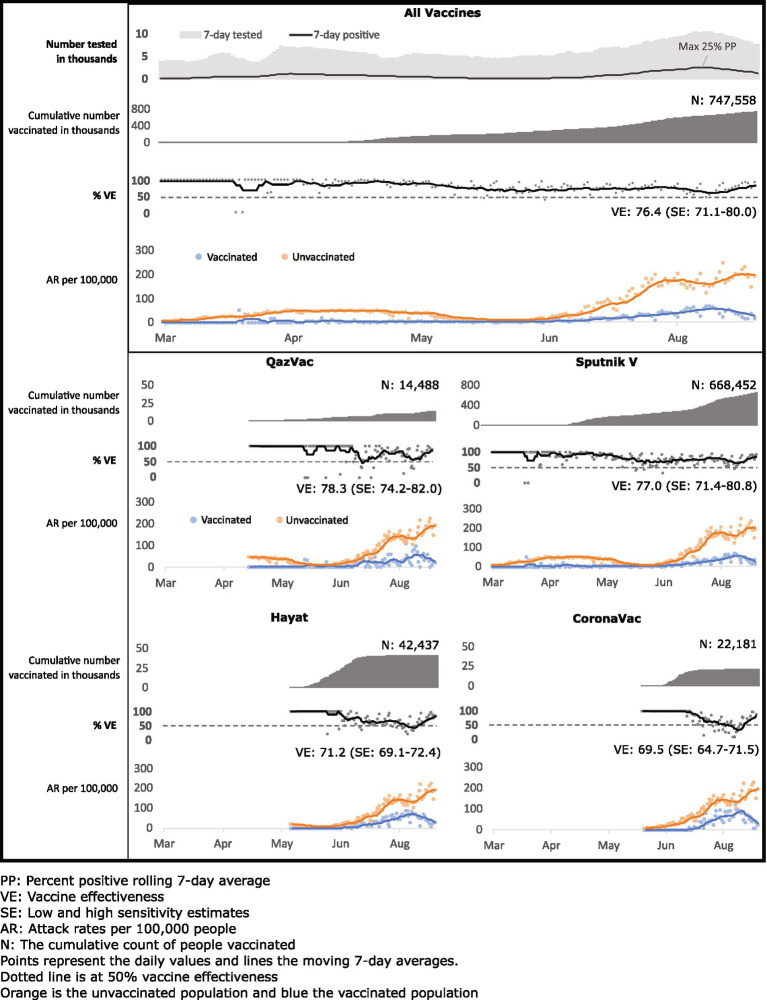
Cumulative vaccinated, vaccine effectiveness against SARS-CoV-2 infection, and attack rates among vaccinated and unvaccinated populations by four COVID-19 vaccines in Almaty, Kazakhstan, 2021.

For all vaccines, the attack rate for COVID-19 per 100,000 was consistently higher over the 7 days moving average for unvaccinated persons than for vaccinated persons (Figure 2). The 7 days moving average of daily vaccine effectiveness for all four vaccines remained above the WHO 50% threshold for vaccine efficacy (5), except for one-week dips for QazVac in June and for CoronaVac and Hayat-Vax in August. Based on overall incidence in the study period, vaccination with these four vaccines prevented 100,213 cases of SARS-CoV-2 breakthrough infection in Almaty.

Among people who were fully vaccinated (*n* = 747,558), the 90 days cumulative hazard of developing a SARS-CoV-2 breakthrough infection was 2.2%, ranging from 2.0% for Sputnik V to 3.5% for Hayat-Vax (Table 3). The cumulative hazard was significantly different by age group. For people 60 years old and older the cumulative hazard was double that of people 18–39 years old at the 180 days (12.8% vs. 5.0%, respectively) and 90-day follow-ups (2.9% vs. 1.9%).

**Table 3:** Cumulative hazard of SARS-CoV-2 breakthrough infection among persons fully vaccinated by four COVID-19 vaccines, Almaty, Kazakhstan, 2021.

Group	Fully vaccinated, *N*	COVID-19 events	Cumulative hazard percent at day						*p* ^a^
			30	60	90	120	150	180	
**All vaccine**	747,558	11,472	0.5%	1.2%	2.2%	3.9%	5.1%	6.7%	Ref
Sputnik	668,452	9,826	0.4%	1.0%	2.0%	3.7%	4.9%	6.5%	<0.001
QazVac	14,488	187	0.5%	1.4%	2.5%	3.5%			<0.001
Hayat	42,437	955	0.4%	2.0%	3.5%				<0.001
CoronaVac	22,181	504	1.0%	2.6%					<0.001
**Age, years**									
18–39	387,024		0.4%	1.1%	1.9%	2.9%	3.8%	5.0%	Ref
40–59	272,225		0.5%	1.2%	2.2%	3.9%	5.2%	6.8%	<0.001
≥60	88,309		0.5%	1.4%	2.9%	5.9%	8.8%	12.8%	<0.001
**Month**									
February	1,171	32	0.1%	0.3%	0.8%	0.9%	1.3%	2.7%	Ref
March	2,844	77	0.3%	0.5%	0.6%	1.0%	2.1%	3.8%	<0.001
April	53,371	1,543	0.1%	0.2%	0.7%	2.3%	3.6%		<0.001
May	147,046	3,805	0.1%	0.3%	1.3%	3.0%			<0.001
June	105,171	2,303	0.3%	1.5%	2.7%				<0.001
July	221,353	3,176	0.8%	2.0%					<0.001
August	208,821	536	0.6%						<0.001
September	7,781	0							

a *p*-value of Wald statistic *z*-test to evaluate whether the beta coefficient of given variable is significantly different from zero using coxph function in R. Model log-likelihood tests, *p* < 0.001.

**Figure 3:**
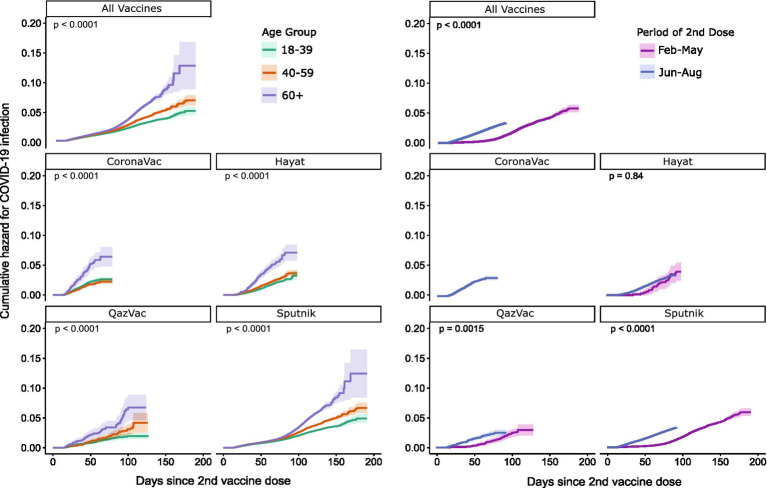
Cumulative hazard curves by age group and days since second dose by vaccine type by four COVID-19 vaccines, Almaty, Kazakhstan, 2021.

This divergence in hazard by age group over follow-up time was observed across the four vaccines (*p* < 0.001) (Figure 3). Cumulative hazard curves began to diverge by age at approximately 90 days postvaccination for Sputnik V, and at approximately 30 days postvaccination for other vaccines. Cumulative hazard is also significantly different by study period (*p* < 0.001) when comparing people who were vaccinated in March to May 2021 to those vaccinated in June to September 2021 (the period when the SARS-CoV-2 Delta variant was active in the region). However, in vaccine-specific analysis, this difference was only significant for QazVac and Sputnik V.

## Discussion

Our study provides a snapshot of vaccine effectiveness in a specific population at specific point in time in the pandemic. We found that at the population-level the four vaccines against COVID-19 used in Kazakhstan were effective at preventing SARS-CoV-2 infection. Vaccination reduced the risk of infection by 76% and prevented over 100,000 cases of SARS-CoV-2 infection in Almaty, the country’s most populous city. The vaccine effectiveness for all four vaccines exceeded the 50% vaccine efficacy minimum threshold set by the WHO for approving vaccines. Vaccine effectiveness also remained relatively stable over the period, despite a slight decrease observed during the months when the SARS-CoV-2 Delta variant became the predominant circulating strain worldwide.

QazVac had 79% vaccine effectiveness in our study but accounted for only 2% of people vaccinated. Vaccine effectiveness for QazVac in our analysis was similar to the vaccine efficacy reported in phase III clinical trials (81%) (10).

It is difficult to compare vaccine effectiveness results across countries, settings and populations because studies are conducted in different phases of the pandemic. Different phases have had different non-pharmaceutical interventions in place and different circulating strains. The predominant vaccine given in Kazakhstan during the period of analysis was Sputnik V (89% of vaccinated people) and our observed vaccine effectiveness of 73% compares to (79% and 86%) vaccine effectiveness from limited observational studies, but lower than 92% vaccine efficacy estimate from phase III clinical trials (24, 25). However, these clinical trials took place before Delta became the predominant variant.

CoronaVac had the lowest vaccine effectiveness in our analysis, and the estimate fell within the range of vaccine efficacy phase III trials (51 to 84%) in several countries (6, 26–29). It is also consistent with vaccine effectiveness against symptomatic disease reported in a comparable study from Chile (66%) (30). Vaccine effectiveness for Hayat-Vax (71%) was also within the range of vaccine effectiveness reported in similar studies in Hungary and Peru (69% and 50%, respectively) (25, 31) and vaccine efficacy reported by Sinopharm (the same BBIBP-CorV vaccine manufactured in China) for phase III clinical trials (79%) (6, 32).

Although COVID-19 vaccines used globally are highly effective, people who are fully vaccinated may experience breakthrough SARS-CoV-2 infections, especially when the incidence is high, and the proportion of people vaccinated in a population is low. In Kazakhstan, population vaccination began shortly before the Delta surge. As expected, breakthrough cases occurred among vaccinated people, increasing as SARS-CoV-2 incidence increased in the country. However, out of every 100 people fully vaccinated in Almaty during our study period less than 2 had breakthrough infections. The low proportion of people getting breakthrough COVID-19 in our study is within the range of that reported in other settings (<1% to 8%) (33, 34). Not only are vaccines effective for reducing infection, but they are also highly effective at reducing morbidity and mortality (35, 36); in our study, the majority (74%) of people with breakthrough infection had mild disease.

Proportion of breakthrough infections increased over time, especially among people ≥60 years of age and during the SARS-CoV-2 Delta variant surge (6, 37). This increase was most notable among people vaccinated with Sputnik V because it had the longest period of follow-up, but the follow-up period was limited to <90 days for two of the four vaccines studied. We cannot distinguish whether the declining effectiveness was attributable to waning immunity or emergence of new SARS-CoV-2 variants. However, waning immunity up to 3 and 3-to-6 months after vaccination has been demonstrated in several studies that found reduced neutralizing antibodies over time in people vaccinated against COVID-19 with CoronaVac, Sputnik V, and BNT162b2 (Pfizer–BioNTech) vaccines, and others (6, 38).

Although the COVID-19 picture has changed and will continue to change, globally we are still experiencing daily cases of COVID-19, including high burden of COVID-19 associated mortality. Vaccination has been proven to be an effective tool in preventing severe illness, hospitalization, and death caused by COVID-19. With the decrease in the incidence rate, it is crucial to continue vaccinating as many people as possible to ensure that the virus does not have a resurgence. It is also critical to continually monitor effectiveness of vaccines during these resurgences. Our study demonstrates a cost-effective way of doing this using data that is already being routinely collected by the Ministry of Health.

During data cleaning, we found 1,204 cases where the dates of vaccination and disease onset conflicted with national vaccine administration directives. While some of these incongruencies could be data entry errors, they could also be from falsification of vaccine records. Falsification of vaccine records was known to be a problem in the Central Asia Region and in Kazakhstan where vaccination was compulsory for certain populations (39–41). Falsification of records undermine efforts to mitigate COVID-19 because vaccines may appear to be less effective than they are.

Vaccine confidence can also be boosted through increased global recognition of efficacious vaccines. Despite trials showing high vaccine efficacy and studies showing real-world vaccine effectiveness in several countries, only one of the four vaccines in our study, CoronaVac, is accepted as proof of vaccination in several countries. The other vaccines, two of which have been used widely globally, have not completed submission of all requirements for WHO approval.

This is the first study from Kazakhstan reporting the real-world effectiveness of fours available vaccines based on the retrospective cohort analysis in the largest city of the country using aggregated vaccination data and individual-level breakthrough case national surveillance data. This is also the first study to estimate vaccine effectiveness for QazVac, an inactivated vaccine developed in Kazakhstan, in real-world conditions. Our results are relevant for planning and scale-up of COVID-19 vaccination efforts in the region.

Our study is subject to at least five important limitations. First, we could not assess vaccine effectiveness against severe disease, hospitalization, and death because individual level data on unvaccinated people who died or were hospitalized were not available for our analysis. Second, we were not able to adjust for potential confounders, such as gender, age or underlying medical conditions, because this data was not available in the reference group. Our analysis is likely not biased by age because the population proportion ages 60 years and older among vaccinated people (12%) approximates (42) that proportion in the general adult population (Appendix B: Supplementary Figure) Additionally, we conducted sensitivity analysis looking at models that adjusts for proportion vaccinated by age group over time and results fell within the high and low sensitivity estimates (Appendix A: Supplementary Table). Third, when only people with symptoms of COVID-19 in the population receive SARS-CoV-2 PCR testing, vaccine effectiveness may be overestimated. However, testing was widely available in Almaty during the study period, with no documented shortages. There were no changes in testing policy over this time, and proof of COVID-19 negative test results were required for participation in several activities. Also, the seven-day moving average of COVID-19 tests tracked the epidemic curve (Figure 2). We did sensitivity analysis accounting for the daily percent positivity for testing, and results fell within our high and low sensitivity estimates. Fourth, individual data was available only for COVID-19 cases among vaccinated people and data for unvaccinated people were aggregate by day and age group (18–39, 40–59, and ≥60). We could not remove potential duplicates from aggregated COVID-19 case data. Fifth, though we attempted to remove fake vaccine passports from the analysis, we cannot ascertain status of all vaccine records and people who were not vaccinated may have been included in the vaccinated population.

## Conclusion

We conducted a retrospective cohort study in Almaty to evaluate effectiveness of Sputnik V, QazVac, Hayat-Vax, and CoronaVac COVID-19 vaccines using aggregated vaccination data and individual-level COVID-19 case data from ongoing surveillance. We found that four vaccines used in Almaty had good effectiveness against SARS-CoV-2 infection vaccine effectiveness of 76.4% (range 69.5% to 78.6% across vaccine types). Vaccine effectiveness studies have known limitations regarding the outcome studied, confounders, and misclassification, however, we minimized the biases in our analysis and interpreted our results considering these limitations. Our results can be used to increase vaccine confidence in Kazakhstan and the region. Scale-up of efforts to fully vaccinate all eligible unvaccinated persons and to provide booster doses to persons already fully vaccinated, people ≥60 years of age and during variant surges, will help reduce SARS-CoV-2 incidence. The methodology used for this analysis can be adopted for ongoing monitoring of vaccine effectiveness nationally and regionally.

## Data availability statement

The data analyzed in this study is subject to the following licenses/restrictions: deidentified data might be made available for authorized researchers after application to the Ministry of Healthcare of the Republic of Kazakhstan. The authors confirm that the manuscript is an honest, accurate, and transparent account of the investigation being reported; and that no important aspects of the study have been omitted. Requests to access these datasets should be directed to hny5@cdc.gov.

## Ethics statement

The study was deemed non-research public health activity by the Ministry of Healthcare of Kazakhstan and the institutional review board of the CDC and conducted in accordance with the CDC policy (project ID # 0900f3eb81e4b259).

## Author contributions

DN, RH, MS, GN, AY, DS, AH, and AT: conceptualization and writing—review and editing. DN, RH, MS, GN, AH, and AY: methodology. DN, RH and GN: software, validation, and formal analysis. DN, RH, AH, and GN: investigation. DN, MS, GN, AY, and AT: resources and data curation. DN, RH, AH, and DS: writing—original draft preparation. DN and RH visualization. AY, DS, and AT: supervision. MS and DN: project administration. All authors contributed to the article and approved the submitted version.

## Funding

None for study implementation. The cost of publication will be funded by the CDC (TEPHINET cooperative agreement # GH000044).

## Conflict of interest

The authors declare that the research was conducted in the absence of any commercial or financial relationships that could be construed as a potential conflict of interest.

## Publisher’s note

All claims expressed in this article are solely those of the authors and do not necessarily represent those of their affiliated organizations, or those of the publisher, the editors and the reviewers. Any product that may be evaluated in this article, or claim that may be made by its manufacturer, is not guaranteed or endorsed by the publisher.

## References

[1] World Health Organization Health Emergency Dashboard. Kazakhstan situation. Data as received by WHO from national authorities by 5:51pm CET. (2021). Available at: https://covid19.who.int/region/euro/country/kz. (Accessed 17 December, 2021)

[2] Over 8 million Kazakhstanis fully vaccinated against coronavirus. (2021). Available at: https://primeminister.kz/en/news/bolee-8-mln-kazahstancev-polnostyu-vakcinirovalis-ot-koronavirusa-30103645. (Accessed 30 November, 2021)

[3] Zahid MN Moosa MS Perna S Buti EB. A review on COVID-19 vaccines: stages of clinical trials, mode of actions and efficacy. Arab J Basic Appl Sci. (2021) 28:225–33. doi: 10.1080/25765299.2021.1903144

[4] World Health Organization. Vaccine efficacy, effectiveness and protection. (2021). Available at: https://www.who.int/news-room/feature-stories/detail/vaccine-efficacy-effectiveness-and-protection. (Accessed 14 July, 2021)

[5] World Health Organization. Considerations for evaluations of COVID-19 vaccines. Points to consider for manufacturers of COVID-19 vaccines. (2020). Version. Available at: https://www.who.int/medicines/regulation/prequalification/prequal-vaccines/WHO_Evaluation_Covid_Vaccine.pdf. (Accessed 23 September, 2020)

[6] Mallapaty S. China’s COVID vaccines have been crucial—now immunity is waning. Nature. (2021) 598:398–9. doi: 10.1038/d41586-021-02796-w, PMID: 34650240

[7] Jones I Roy P. Sputnik V COVID-19 vaccine candidate appears safe and effective. Lancet. (2021) 397:642–3. doi: 10.1016/S0140-6736(21)00191-4, PMID: 33545098 PMC7906719

[8] Logunov DY Dolzhikova IV Shcheblyakov DV Tukhvatulin AI Zubkova OV Dzharullaeva AS. Safety and efficacy of an rAd26 and rAd5 vector-based heterologous prime-boost COVID-19 vaccine: an interim analysis of a randomised controlled phase 3 trial in Russia. Lancet. (2021) 397:671–81. doi: 10.1016/S0140-6736(21)00234-8, PMID: 33545094 PMC7852454

[9] Zakarya K Kutumbetov L Orynbayev M Abduraimov Y Sultankulova K Kassenov M. Safety and immunogenicity of a QazCovid-in^®^ inactivated whole-virion vaccine against COVID-19 in healthy adults: a single-centre, randomised, single-blind, placebo-controlled phase 1 and an open-label phase 2 clinical trials with a 6 months follow-up in Kazakhstan. EClinicalMedicine. (2021) 39:101078. doi: 10.1016/j.eclinm.2021.101078 34414368 PMC8363482

[10] Khairullin B Zakarya K Orynbayev M Abduraimov Y Kassenov M Sarsenbayeva G. Efficacy and safety of an inactivated whole-virion vaccine against COVID-19, QazCovid-in^®^, in healthy adults: a multicentre, randomised, single-blind, placebo-controlled phase 3 clinical trial with a 6-month follow-up. EClinicalMedicine. (2022) 50:101526. doi: 10.1016/j.eclinm.2022.101526, PMID: 35770251 PMC9233449

[11] World Bank, United Nations, Census, GeoNames. Almaty, Kazakhstan Population. (2022). Available at: https://populationstat.com/kazakhstan/almaty.

[12] Arbel R Sergienko R Friger M Peretz A Beckenstein T Yaron S. Effectiveness of a second BNT162b2 booster vaccine against hospitalization and death from COVID-19 in adults aged over 60 years. Nat Med. (2022) 28:1486–90. doi: 10.1038/s41591-022-01832-0, PMID: 35468276

[13] Hammerman A Sergienko R Friger M Beckenstein T Peretz A Netzer D. Effectiveness of the BNT162b2 vaccine after recovery from Covid-19. N Engl J Med. (2022) 386:1221–9. doi: 10.1056/NEJMoa2119497, PMID: 35172072 PMC8908846

[14] Tang L Zhang Y Wang F Wu D Qian Z-H Zhang R. Relative vaccine effectiveness against Delta and Omicron COVID-19 after homologous inactivated vaccine boosting: a retrospective cohort study. BMJ Open. (2022) 12:e063919. doi: 10.1136/bmjopen-2022-063919, PMID: 36368753 PMC9659710

[15] Nunes B Rodrigues AP Kislaya I Cruz C Peralta-Santos A Lima J. mRNA vaccine effectiveness against COVID-19-related hospitalisations and deaths in older adults: a cohort study based on data linkage of national health registries in Portugal, February to August 2021. Eur Secur. (2021) 26:2100833. doi: 10.2807/1560-7917.ES.2021.26.38.2100833 PMC8462036 34558406

[16] Therneau T Crowson C Clinic M. Using time dependent covariates and time dependent coefficients in the Cox model. (2023). Available at: https://cran.r-project.org/web/packages/survival/vignettes/timedep.pdf

[17] Andersen PK Gill RD. Cox’s regression model for counting processes: a large sample study. Ann Stat. (1982) 10:1100–20. doi: 10.1214/aos/1176345976

[18] Therneau T.M. Grambsch P.M. (2000) Modeling survival data: extending the Cox model. Berlin. Springer

[19] Therneau T (2021). _A package for survival analysis in R_. R package version 3.2-13. Available at: https://CRAN.R-project.org/package=survival.

[20] Cox DR. Analysis of survival data. 1st ed Chapman and Hall/CRC (1984).

[21] Cox DR. Regression models and life-tables. J R Stat Soc B. (1972) 34:187–202. doi: 10.1111/j.2517-6161.1972.tb00899.x

[22] World Health Organization. Evaluation of COVID-19 vaccine effectiveness in a changing landscape of COVID-19 epidemiology and vaccination: interim guidance, 1 October 2022: second addendum to evaluation of COVID-19 vaccine effectiveness: interim guidance. Geneva: World Health Organization (2022) Contract No.: WHO/2019-nCoV/vaccine_effectiveness/VE_evaluations/2022.1.

[23] World Health Organization. Evaluation of COVID-19 vaccine effectiveness: interim guidance, 17 March 2021. Geneva: World Health Organization (2021) Contract No.: WHO/2019-nCoV/vaccine_effectiveness/measurement/2021.1.

[24] González S Olszevicki S Salazar M Calabria A Regairaz L Marín L. Effectiveness of the first component of gam-COVID-Vac (Sputnik V) on reduction of SARS-CoV-2 confirmed infections, hospitalisations and mortality in patients aged 60–79: a retrospective cohort study in Argentina. EClinicalMedicine. (2021) 40:101126. doi: 10.1016/j.eclinm.2021.101126, PMID: 34541480 PMC8435263

[25] Vokó Z Kiss Z Surján G Surján O Barcza Z Pályi B. Nationwide effectiveness of five SARS-CoV-2 vaccines in Hungary-the HUN-VE study. Clin Microbiol Infect (2022);28:398–404. doi: 10.1016/j.cmi.2021.11.011 34838783 PMC8612758

[26] de Faria E Guedes AR Oliveira MS de Godoy Moreira MV Maia FL dos Santos Barboza A. Performance of vaccination with CoronaVac in a cohort of healthcare workers (HCW)—preliminary report. *medRxiv* (2021). Available at: https://www.medrxiv.org/content/10.1101/2021.04.12.21255308v1

[27] World Health Organization. Evidence assessment: Sinovac/CoronaVac COVID-19 vaccine. For recommendation by the strategic advisory group of experts (Sage) on immunization. (2021). Available at https://cdn.who.int/media/docs/default-source/immunization/sage/2021/april/5_sage29apr2021_critical-evidence_sinovac.pdf. (Accessed April 29, 2021)

[28] Fadlyana E Rusmil K Tarigan R Rahmadi AR Prodjosoewojo S Sofiatin Y. A phase III, observer-blind, randomized, placebo-controlled study of the efficacy, safety, and immunogenicity of SARS-CoV-2 inactivated vaccine in healthy adults aged 18–59 years: An interim analysis in Indonesia. Vaccine. (2021) 39:6520–8. doi: 10.1016/j.vaccine.2021.09.052, PMID: 34620531 PMC8461222

[29] Tanriover MD Doğanay HL Akova M Güner HR Azap A Akhan S. Efficacy and safety of an inactivated whole-virion SARS-CoV-2 vaccine (CoronaVac): interim results of a double-blind, randomised, placebo-controlled, phase 3 trial in Turkey. Lancet. (2021) 398:213–22. doi: 10.1016/S0140-6736(21)01429-X, PMID: 34246358 PMC8266301

[30] Jara A Undurraga EA González C Paredes F Fontecilla T Jara G. Effectiveness of an inactivated SARS-CoV-2 vaccine in Chile. N Engl J Med. (2021) 385:875–84. doi: 10.1056/NEJMoa2107715, PMID: 34233097 PMC8279092

[31] Silvia-Valencia J Soto-Becerra P Escobar-Agreda S Fernández-Navarro M Moscoso-Porras M Solari L. (2021). Efectividad de la vacuna BBIBP-CorV para prevenir infección y muerte en personal de salud [effectiveness of the BBIBP-CorV vaccine to prevent infection and death in health personnel] Technical Report. (in Spanish). Tabla 4, figura 2. Ministry of Health of Peru.

[32] al Kaabi N Zhang Y Xia S Yang Y Al Qahtani MM Abdulrazzaq N. Effect of 2 inactivated SARS-CoV-2 vaccines on symptomatic COVID-19 infection in adults: a randomized clinical trial. JAMA. (2021) 326:35–45. doi: 10.1001/jama.2021.8565, PMID: 34037666 PMC8156175

[33] News CTV. What do we know about breakthrough COVID-19 cases? Experts break down the science. (2021) Available at: https://www.ctvnews.ca/health/coronavirus/what-do-we-know-about-breakthrough-covid-19-cases-experts-break-down-the-science-1.5561879. (Accessed August 26, 2021)

[34] New York State Department of Health. COVID-19 breakthrough data. Current estimates of cases and hospitalizations by vaccine status, and vaccine effectiveness. (2021) Available at: https://coronavirus.health.ny.gov/covid-19-breakthrough-data.

[35] Team CC-VBCI. COVID-19 vaccine breakthrough infections reported to CDC—United States, January 1–April 30, 2021. MMWR Morb Mortal Wkly Rep. (2021) 70:792–3. doi: 10.15585/mmwr.mm7021e3 34043615 PMC8158893

[36] Agrawal U Katikireddi SV McCowan C Mulholland RH Azcoaga-Lorenzo A Amele S. COVID-19 hospital admissions and deaths after BNT162b2 and ChAdOx1 nCoV-19 vaccinations in 2·57 million people in Scotland (EAVE II): a prospective cohort study. Lancet Respir Med. (2021) 9:1439–49. doi: 10.1016/S2213-2600(21)00380-5, PMID: 34599903 PMC8480963

[37] Levin EG Lustig Y Cohen C Fluss R Indenbaum V Amit S. Waning immune humoral response to BNT162b2 Covid-19 vaccine over 6 months. N Engl J Med. (2021) 385:e84. doi: 10.1056/NEJMoa2114583, PMID: 34614326 PMC8522797

[38] Lapa D Grousova DM Matusali G Meschi S Colavita F Bettini A. Retention of neutralizing response against SARS-CoV-2 Omicron variant in Sputnik V-vaccinated individuals. Vaccines. (2022) 10:817. doi: 10.3390/vaccines10050817 35632574 PMC9144866

[39] Kazakhstan is awash in fake vaccination passports. (2021). Available at: https://www.economist.com/asia/2021/07/24/kazakhstan-is-awash-in-fake-vaccination-passports. (Accessed July 24 2021)

[40] Greig Jonathan, Staff writer, price for fake COVID-19 vaccine cards and passports drops to $100: report. ZDNet Security (2021). Available at: https://www.zdnet.com/article/price-for-fake-covid-19-vaccine-cards-and-passports-drops-to-100-report/

[41] Hanna Belovolchenko, Coronavirus vaccines and Covid passports are being sold on the dark web: Zaborona reports on cost and availability in Ukraine. (2021). Available at: https://zaborona.com/en/coronavirus-vaccines-and-covid-passports-are-being-sold-on-the-dark-web-zaborona-reports-on-cost-and-availability-in-ukraine/

[42] World Bank staff estimates based on age/sex distributions of United Nations population division’s world population prospects: 2019 revision. Population ages 65 and above (% of total population)—Kazakhstan. Available at: https://data.worldbank.org/indicator/SP.POP.65UP.TO.ZS?locations=KZ.

