# Supplementary material for: Effectiveness of four vaccines in preventing SARS-CoV-2 infection in Almaty, Kazakhstan in 2021: retrospective population-based cohort study
Source: Front Public Health. 2023 Jun 7;11:1205159. doi: 10.3389/fpubh.2023.1205159 (PMC10282771; doi:10.3389/fpubh.2023.1205159)
Supplement: Supplementary file 2 [file Table_1.docx]

**Appendix a:**

**Supplement Table**

Sensitivity analysis of vaccine effectiveness of four vaccines in preventing COVID-19 in Almaty, Kazakhstan, 2021

| **Start of person-time contribution** | **Vaccine** | **Model 1** | | **Model 2** | | **Model 3** | |
| --- | --- | --- | --- | --- | --- | --- | --- |
|  |  | **% VE** | **95% CI** | **% VE** | **95% CI** | **% VE** | **95% CI** |
| Vaccine 2 | Any vaccine | 76.4 | (75.9-76.9) | 75.9 | (75.4-76.4) | 75.9 | (75.4-76.4) |
|  | CoronaVac | 69.5 | (66.7-72.0) | 69.1 | (66.2-71.7) | 65.5 | (62.3-68.4) |
|  | Hayat | 71.2 | (69.3-73.0) | 70.8 | (68.9-72.6) | 67.2 | (65.0-69.2) |
|  | QazVac | 78.6 | (75.3-81.4) | 78.2 | (74.9-81.1) | 75.2 | (71.4-78.5) |
|  | Sputnik | 77 | (76.5-77.5) | 76.5 | (75.9-77.0) | 73.8 | (73.2-74.4) |
| Vaccine 2 - 14 days (high estimate) | Any vaccine | 80 | (79.6-80.4) | 79.4 | (79.0-79.8) | 76.9 | (76.4-77.3) |
|  | CoronaVac | 71.5 | (68.9-73.9) | 71 | (68.3-73.4) | 67.7 | (64.7-70.4) |
|  | Hayat | 72.4 | (70.6-74.1) | 71.9 | (70.1-73.7) | 68.5 | (66.5-70.5) |
|  | QazVac | 82 | (79.2-84.4) | 81.6 | (78.8-84.1) | 79.1 | (75.9-81.9) |
|  | Sputnik | 80.8 | (80.4-81.2) | 80.2 | (79.7-80.6) | 77.7 | (77.2-78.2) |
| Vaccine 2 + 7 days | Any vaccine | 74.2 | (73.6-74.7) | 73.7 | (73.1-74.2) | 70.8 | (70.2-71.4) |
|  | CoronaVac | 67.8 | (64.9-70.5) | 67.4 | (64.5-70.2) | 63.8 | (60.5-66.9) |
|  | Hayat | 70.5 | (68.5-72.3) | 70.1 | (68.1-71.9) | 66.4 | (64.1-68.4) |
|  | QazVac | 76.6 | (72.9-79.7) | 76.2 | (72.6-79.4) | 73 | (68.8-76.6) |
|  | Sputnik | 74.7 | (74.1-75.2) | 74.2 | (73.6-74.7) | 71.4 | (70.7-72.0) |
| Vaccine 2 + 14 days (low estimate) | Vaccinated | 71.1 | (70.5-71.6) | 70.6 | (70.0-71.2) | 67.5 | (66.8-68.1) |
|  | CoronaVac | 64.7 | (61.5-67.7) | 64.4 | (61.2-67.4) | 60.7 | (57.0-64.0) |
|  | Hayat | 69.1 | (67.1-71.0) | 68.7 | (66.7-70.7) | 65 | (62.6-67.1) |
|  | QazVac | 74.2 | (70.2-77.7) | 73.9 | (69.9-77.4) | 70.4 | (65.8-74.4) |
|  | Sputnik | 71.4 | (70.8-72.1) | 70.9 | (70.3-71.6) | 67.9 | (67.2-68.6) |

VE: Vaccine effectiveness, CI: Confidence interval

Model 1: Time-adjusted

Model 2: Adjusted for time, daily cumulative proportion vaccinated ≥60 years old

Model 3: Adjusted for time, daily cumulative proportion vaccinated ≥60 years old, and daily COVID-19 positive test rate
